# Diagnostic value of atrial natriuretic peptide (ANP), B-type natriuretic peptide (BNP) and their correlation with lipoproteins in dogs with myxomatous mitral valve disease

**DOI:** 10.1186/s12917-022-03548-2

**Published:** 2022-12-23

**Authors:** Zohreh Khaki, Parviz Nooshirvani, Darioush Shirani, Majid Masoudifard

**Affiliations:** 1 Department of Clinical Pathology, Faculty of Veterinary Medicine, University of Tehran, Qareeb St., Azadi Ave, Tehran, Iran; 2 Department of Internal Medicine, Faculty of Veterinary Medicine, University of Tehran, Tehran, Iran; 3 Department of Surgery and Radiology, Faculty of Veterinary Medicine, University of Tehran, Tehran, Iran

**Keywords:** Myxomatous mitral valve disease, B-type natriuretic peptide, Atrial natriuretic peptide, Dyslipidemia, Dog

## Abstract

**Background:**

Myxomatous mitral valve disease (MMVD) is the most common diagnosed cardiovascular disease in dogs. Atrial natriuretic peptide (ANP) and B-type natriuretic peptide (BNP) tests are used to diagnose congestive heart failure, but there are conflicting reports about their value in diagnosing the subclinical stages of MMVD in dogs. Moreover, the diagnostic value of blood lipoproteins in dogs with MMVD is still unclear. The purpose of this study was to assess the serum concentrations of ANP, BNP and lipoproteins of dogs with MMVD and to evaluate the correlation of the levels of ANP and BNP with lipoproteins.

**Results:**

This study was performed on 24 dogs with MMVD and 10 healthy dogs. Dogs with MMVD were classified in to stages B1 (*n* = 11), B2 (*n* = 6), C (*n* = 4) and D (*n* = 3) groups according to the classification suggested by American College of Veterinary Internal Medicine guidelines. Our results showed that the mean serum BNP levels were significantly increased for all MMVD groups compared to control dogs. The mean serum ANP levels for the stage B2, C and D groups were significantly higher than the control group, while the mean serum ANP concentrations did not differ significantly between the stage B1 and control groups. An increase in BNP level was observed in 87.5% of patients. Although BNP concentrations were elevated in 100% of dogs with stages C, D and B2, high BNP was observed in 72.72% of dogs with stage B1. Regarding ANP, 58.33% of patients had an increase in ANP. However, elevated ANP levels were found in only 27.27% of patients in stage B1, while increased ANP levels were observed in 66.66 and 100% of patients in stage B2 and C/D groups respectively. Also, in all patients with MMVD, the mean serum concentrations of high-density lipoprotein cholesterol (HDL-C) were approximately 1.7 to 2 times significantly lower than the control group. Additionally, the mean serum low-density lipoprotein cholesterol (LDL-C) increased significantly (1.9-2.7 times) compared to the control group.

There was a significant inverse correlation between HDL-C and BNP, and HDL-C and ANP. LDL-C showed a significant positive correlation with BNP, and ANP. Also, LDL-C, but not HDL-C, had a significant positive correlation with LA/AO ratio, LVIDd, LVIDdN and VHS. BNP and ANP showed a significant positive correlation with LA/AO, LVIDd, LVIDdN and VHS.

**Conclusions:**

Serum BNP has a greater diagnostic value than serum ANP in dogs with MMVD. In addition, serum BNP can be used to determine the subclinical stages of B1 and B2 MMVD. This study also suggests that dogs with subclinical MMVD, showed an increase in BNP along with a decrease in HDL-C and an increase in LDL-C, which are known to be risk factors for cardiovascular diseases in human. However, it seems that high LDL-C is more involved in the pathogenesis of MMVD than low HDL-C. Therefore, periodic testing of serum lipoproteins is recommended in high-risk patients, even if total cholesterol levels are normal.

## Background

Myxomatous mitral valve disease (MMVD) is the most common diagnosed cardiovascular disease in dogs, and has been estimated to account for more than 70% of heart diseases in dogs [1]. MMVD also called as endocardiosis and chronic or degenerative valvular disease [2]. Most dog breeds at elevated risk for MMVD are small to medium sized breeds [3]. The disease is chronic and progressive and the first sign of MMVD is usually a heart murmur, developing after the age of six [1]. MMVD is staged by the American College of Veterinary Internal Medicine (ACVIM). MMVD staging helps the patient not experience more advanced stages of the disease and heart failure with timely treatment. This classification is marked as stage B1 (no clinical signs or evidence of cardiac enlargement), stage B2 (no clinical signs with evidence of cardiac enlargement), stage C (history of clinical signs of congestive heart failure (CHF)) and stage D (end-stage MMVD) [2].

Natriuretic peptides (NPs) include atrial natriuretic peptide) ANP) and B-type natriuretic peptide. These peptide hormones are primarily synthesized by the heart and released into the blood circulation in response to atrial and ventricular overload as well as by neurohumoral stimuli [4]. ANP is synthesized as pre-pro-hormone and then cleaved to pro-hormone and finally to ANP (biologically active peptide). BNP is released by the same mechanism as ANP [5]. ANP and BNP have natriuretic (increased sodium excretion), diuretic (increased fluid excretion) and vasodilating effects. Natriuretic peptides serve as a counter-regulatory system for the renin-angiotensin-aldosterone system. Thus, decrease renin release, thereby decreasing circulating levels of aldosterone. These actions lead to antagonistic effects against angiotensin II [6], and subsequently further natriuresis and diuresis [5]. Therefore, ANP and BNP protect the cardiovascular system from volume overload by their actions [7].

Hyperlipidemia is common in dogs, because there are many changes in the diet and lifestyle of dogs today. Research has shown that a dog’s diet significantly affects lipid metabolism especially lipoproteins involved in cholesterol plasma transport [8]. Moreover hyperlipidemia may be an important emergency in human because of its potential complications such as cardiovascular diseases [9]. In this regard, although research has shown that cardiovascular diseases such as mitral valve disease [1], and atherosclerosis [10] are also common in dogs, the relationship between hyperlipidemia and cardiovascular diseases has not been determined. This could be due to the fact that, unlike humans, canine hyperlipidemia has been considered a relatively benign condition [9]. However, it seems that using the term dyslipidemia or dyslipoproteinemia is much better than hyperlipidemia, because the term hyperlipidemia refers only to an increase in blood cholesterol or triglyceride levels, but the term dyslipidemia describes an increase or decrease in the concentration of lipids in the blood as well as any abnormalities in the properties of lipids and/or lipoproteins [9]. The lipid panel includes measurements of total cholesterol, triglyceride, high-density lipoprotein cholesterol (HDL-C) and low-density lipoprotein cholesterol (LDL-C). Lipid panel evaluation is used to diagnose dyslipidemia, decide the most appropriate therapy, and monitor the effectiveness of treatment, in humans [11]. Lipoproteins, especially HDL-C and LDL-C, are important predictors of cardiovascular disease in humans [8]. In contrast to HDL-C, it has been demonstrated that when cholesterol and LDL-C levels are high, the incidence and prevalence of coronary heart disease are also high [11]. Also, very low-density lipoprotein (VLDL) is a type of lipoprotein. VLDL is estimated by one fifth of triglycerides [12]. However, the clinical and diagnostic value of blood lipoproteins in dogs with MMVD is still unclear. To the best of our knowledge, there is no report on the importance of dyslipidemia and its correlation with ANP and BNP in dogs with MMVD. This study aimed to assess the serum concentrations of ANP, BNP, and lipid panel of dogs with MMVD and to evaluate the correlation of the levels of ANP and BNP with lipoproteins.

## Methods

The study population were referred to the Veterinary Teaching Hospital of the University of Tehran. All dogs were owned by clients and owner consent was obtained for each dog before enrolling in this study. This study was conducted in accordance with the animal ethical guidelines of the faculty of veterinary medicine, University of Tehran, Tehran, Iran (No: 7508017.6.24).

Twenty four dogs were selected with MMVD by clinical examination, auscultation, thoracic radiography and echocardiography. Table 1 shows the breed included in this study. The average age and weight were respectively 9.52 ± 0.68 year and 7.32 ± 0.64 kg. Routine diagnostic laboratory examinations including complete blood count, serum biochemistry profile and thyroid profile (total thyroxine(T4), free T4 concentration and canine thyroid stimulating hormone) were performed to evaluate the possibility of a secondary cause of hyperlipidemia (eg, hypothyroidism, hyperadrenocorticism, diabetes mellitus). However, patients with MMVD and one or more concurrent systemic diseases (such as endocrine, hepatic, renal and pulmonary disease), inflammatory disease and malignances were excluded from the study. Additionally, dogs that had received glucocorticosteroids or any medication affecting lipid metabolism for at least 2 months prior to sampling were excluded. Also, 10 healthy dogs (as control group) of the same age and weight of the patient group were selected. All examinations that were performed on the patients were done on these cases.

**Table 1:** Breed distribution in the MMVD and control groups

Breed	MMVD group	Control group
Terrier	8	4
Terrier mix	4	2
poodle (Toy)	4	1
Japanese spitz	3	1
Pekingese	3	1
Pomeranian	2	1

Blood samples (approximately 3.5 mL) were collected from the cephalic vein into tubes without anticoagulant for serum separation and biochemical assessment. To prepare serum, the blood samples were allowed to clot (60 min, at room temperature), after centrifugation at 2500 rpm for 15 min, serum was transferred in single tubes and kept at − 20 °C until analyzed. Serum levels of BNP and ANP were determined with commercially available canine-specific ELISA kits (Shanghaicrystal Day Biotech Co., LTD; China). Cholesterol and triglyceride concentrations were measured by commercial kits (Pars Azmoon, Tehran, Iran) using an auto analyzer (Elitech selectra prom, France). HDL-C was measured by precipitation of apo B-containing lipoprotein with manganese-heparin and measurement of cholesterol in the supernatant (Pars Azmoon kit, Iran). VLDL was estimated by one fifth of triglyceride [12], and LDL-C was calculated by Friedewald’s formula: LDL-C = TC-(HDL-C + VLDL) [13].

It should be noted that all assays were performed by laboratory technicians who were unaware of the history and diagnosis of dogs. All tests repeated 3 times.

### Radiography and echocardiography

Chest radiographs were taken under a standardized protocol for dogs with a CR system (Kodak, Carestream, USA) to assess the size of the heart using Vertebral Heart Score (VHS) method, and examine the lung field for possible involvement due to heart failure.

Echocardiography (B-mode, M-Mode and Doppler) was then performed to obtain cardiac indices and abnormalities such as valvular and cardiac chamber changes, by an echocardiographic machine (GE-Vivid 7, USA) and a 4-8 MHz phased array transducer. The Color Doppler technique examined blood flow status through the heart and large arteries (aortic and pulmonary arteries). Regurgitation of the cardiac valves (mitral and tricuspid) was determined and valve insufficiency was confirmed in the presence of abnormal return blood flow. Pulse Doppler echocardiography measured the speed, direction and rate of blood flow through the mitral, tricuspid, aortic and pulmonary valves. Left atrium (LA) and Proximal aorta (AO) diameters were measured by two-dimensional short axis echocardiography and then the LA/Ao ratio was measured. Left ventricular internal dimension in diastole (LVIDd) was measured in M-mode. Also, LVIDd: body weight (LVIDdN) was quantified by formula [2]: LVIDdN = LVIDd (cm)/weight (kg) ^0.294^.

### Statistical analysis

Statistical analysis was performed using the commercial software SPSS statistics Version 24.0. Normal distribution of the data was confirmed by the Kolmogorov-Smirnov test. Thus, data were analyzed by one-way ANOVA, and then post hoc multiple comparisons were made using LSD. Also, the correlations between variables were analyzed by Pearson test to measure the strength and direction of the linear correlation. A *P*-value of less than0.05 was considered statistically significant. All values are expressed as mean ± standard error (SE).

## Results

This study was performed on 24 dogs with MMVD and 10 healthy dogs as control group. Dogs with MMVD were classified in to stages B1 (*n* = 11), B2 (*n* = 6), C (*n* = 4) and D (*n* = 3) groups according to the classification suggested by American College of Veterinary Internal Medicine (ACVIM) [2]. Physical examination, radiographic, and echocardiographic findings of dogs with MMVD and the control group are shown in Table 2. Dogs in stages B1 and B2 had no clinical signs, but dogs in stages C and D had clinical signs or a history of congestive heart failure (including dyspnea and radiographic evidence of pulmonary infiltrates).

**Table 2:** Physical examination, radiographic, and echocardiographic findings of dogs with MMVD and control group

	Control group Mean ± SE (min- max)	Stages of MMVD group Mean ± SE (min- max)			
		B1	B2	C	D
Heart rate (bpm)	126.33±4.96a (104-140)	127.64 ± 3.75 a (108-160)	129.17 ± 4.90a (120-150)	158.50 ± 7.45b (146-180)	163.33 ± 6.00b (155-175)
Murmur intensity	No	No / < 3/6	≥3/6	> 3/6	> 3/6
LA:AO ratio	1.27 ± 0.02 a (1.20-1.37)	1.30 ± 0.04a (1.06-1.5)	1.67 ± 0.03b (1.6-1.8)	2.07 ± 0.21c (1.7-2.68)	2.46 ± 0.13 d (2.2-2.60)
LVIDd (mm)	19.96 ± 0.92 a (18-23)	25.24 ± 2.28 a (16-41)	28.17 ± 1.88a (21-33)	31.20 ± 2.73 a (27-39)	44.20 ± 13.11b (30-70)
LVIDdN (cm/kg^0.294^)	1.09 ± 0.04 a (1.03-1.22)	1.31 ± 0.07 b (0.76-1.56)	1.59 ± 0.09 b (1.35-1.90)	1.76 ± 0.15 c (1.49-2.22)	2.52 ± 0.7 c (1.77-3.97)
VHS	9.7 ± 0.18 a (9.4-10.3)	9.99 ± 0.1a (9.5-10.5)	10.10 ± 0.07 a (9.9-10.4)	11.2 ± 0.27 b (10.8-12)	11.53 ± 0.14 b (11.3-11.8)
Radiographic evidence of pulmonary infiltrates	No	No	No	Yes	Yes

Dogs in stage B1 had LA: AO < 1.6 and LVIDdN < 1.7 cm/kg^0.294^. Dogs in stage B2 had LA: Ao ≥ 1.6 or LVIDdN≥1.7 cm/kg^0.294^

Different letters show significant difference (*p* < 0.05). *MMVD* Myxomatous mitral valve disease, *LA/AO* Left Atrium/aorta ratio, *LVIDd* Left ventricular internal dimension-diastole, *LVIDdN* LVIDd normalized for body weight; VHS, Vertebral heart score

**Fig. 1:**
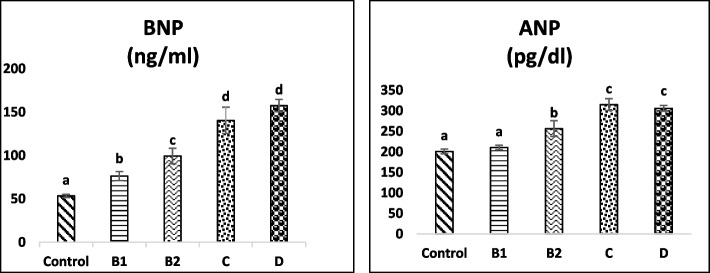
Comparison between stages of MMVD group and control in serum BNP, ANP. Data are expressed as mean ± SE. Different letters show significant differences, *P* < 0.05 MMVD, myxomatous mitral valve disease; BNP, B-type natriuretic peptide; ANP, atrial natriuretic peptide.

Figure 1 shows the serum concentration of BNP and ANP in dogs with MMVD and control group. The mean serum BNP levels for the stage B1 (*p* = 0.01), stage B2 (*p* = 0.000), stage C (*p* = 0.000) and stage D (*p* = 0.000) groups were significantly greater than the mean serum BNP levels for the control group. The increase in BNP concentration in stages B1, B2 and C/D groups was 0.5, less than one and 2.9 times higher than in the control group, respectively. Also the mean serum BNP levels for the stage C group were significantly greater than the mean serum BNP levels for the stage B1 (*p* = 0.000) and B2 groups (*p* = 0.001). Additionally, dogs with stage D of MMVD had significantly greater mean serum BNP levels than dogs with stages B1 and B2 (*p* = 0.000). The mean serum BNP levels for the stage B2 group were meaningfully higher than the mean serum BNP levels for the stage B1 group (*p* = 0.008). There was no significant difference in serum BNP levels between stage C and D groups.

Regarding ANP, there were no meaningful differences between stage B1 and healthy groups. In contrast, the mean serum ANP concentrations for stage B2 (*p* = 0.001), stage C (*p* = 0.000) and stage D (*p* = 0.000) groups were significantly higher than the mean serum ANP levels for the control group. The increase in ANP levels in the stage C and D groups was more significant (approximately 1.55 times) than in the control group. Dogs with stage C of MMVD had significantly greater mean serum ANP levels than dogs with stages B1 (*p* = 0.000) and B2 (*p* = 0.002). In addition, the mean serum ANP concentrations for stage D group was significantly higher than the mean serum ANP levels for stages B1 (*p* = 0.000) and B2 (*p* = 0.01) groups. Also, the mean serum ANP levels for the stage B2 group were significantly higher than the mean serum ANP levels for the stage B1 group (*p* = 0.007) (Fig. 1). However, there was no significant difference in serum ANP levels between stage C and D groups (*p* > 0.05).

**Fig. 2:**
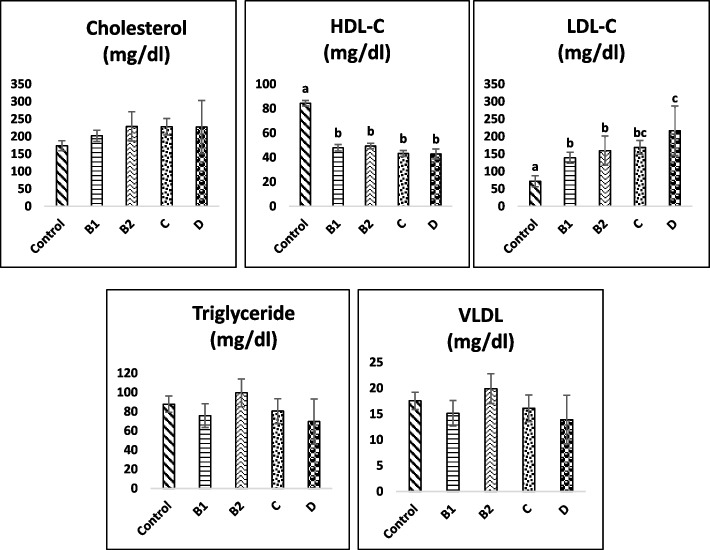
Comparison between stages of MMVD group and control in serum cholesterol, HDL-C, LDL-C, triglyceride and VLDL. Different letters show significant differences, *P* < 0.05 MMVD, myxomatous mitral valve disease; HDL-C, High-density lipoprotein; LDL-C, Low-density lipoprotein; VLDL, Very low-density lipoprotein.

Figure 2 shows the serum concentration of lipoproteins in dogs with MMVD and control groups. Although, the mean serum cholesterol levels for the stages of MMVD group were increased, there was no significant difference between different stages of MMVD and control group. Also, in all patients with MMVD, the mean serum concentrations of high-density lipoprotein cholesterol (HDL-C) were approximately 1.7 to 2 times significantly lower than the control group. Additionally, the mean serum low-density lipoprotein cholesterol (LDL-C) increased significantly (1.9-2.7 times) compared to the control group. Our results showed that dogs in the stage D group had the lowest HDL-C level and the highest LDL-C level compared to other groups. Dogs with stage D of MMVD had significantly greater mean serum LDL-C levels than dogs with stages B1 (*p* = 0.03) and B2 (*p* = 0.02). However, there were no significant differences in the mean serum HDL-C concentrations in dogs with different stages of MMVD (*p* > 0.05). Also, our findings showed that no significant changes were observed in serum VLDL and triglyceride levels (Fig. 2). The results of the correlation analysis are shown in Table 3. The present study showed a significant positive correlation between serum cholesterol concentrations and BNP(*r* = 0.366, *p* = 0.033). Moreover, we found a significant inverse correlation between serum concentrations of HDL-C and BNP (*r* = − 0.62, *p* = 0.000), and serum concentrations of HDL-C and ANP(*r* = − 0.510, *p* = 0.002). Also, serum LDL-C showed a significant positive correlation with BNP (*r* = 0.503, *p* = 0.002), ANP (*r* = 0.413, *p* = 0.015), LA/AO ratio (*r* = 0.516, *p* = 0.004), LVIDd (*r* = 0.689, *p* = 0.000), LVIDdN (*r* = 0.660, *p* = 0.000) and VHS (*r* = 0.584, *p* = 0.001). A significant positive correlation was found between serum cholesterol levels and LA/AO ratio (*r* = 0.455, *p* = 0.013), serum cholesterol levels and LVIDd (*r* = 0.628, *p* = 0.000), serum cholesterol levels and LVIDdN (*r* = 0.606, *p* = 0.001) and serum cholesterol levels and VHS (*r* = 0.492, *p* = 0.008). Serum BNP levels showed a significant positive correlation with LA/AO(*r* = 0.836, *p* = 0.000), LVIDd (*r* = 0.510, *p* = 0.005), LVIDdN (*r* = 0.564, *p* = 0.002) and VHS (*r* = 0.813, *p* = 0.000). Additionally, there were a significant positive correlation between serum ANP levels and LA/AO(*r* = 0.770, *p* = 0.000), serum ANP levels and LVIDd (*r* = 0.457, *p* = 0.013), serum ANP levels and LVIDdN (*r* = 0.548, *p* = 0.003) and serum ANP levels and VHS (*r* = 0.760, *p* = 0.000). No other correlations were identified between variables.

**Table 3:** Results of correlation analysis

		BNP	ANP	Cholesterol	HDL-C	LDL-C	Triglyceride	VLDL
LA/Ao	r	.836^a^	.770^a^	.455^b^	−.361	.516^a^	.050	.050
	p	.000	.000	.013	.054	.004	.796	.796
LVIDd	r	.510^a^	.457^b^	.628^a^	-.414^b^	.689^a^	.067	.067
	p	.005	.013	.000	.025	.000	.729	.729
LVIDdBW	r	.564^a^	.548^a^	.606^a^	-.407^b^	.660^a^	.169	.169
	p	.002	.003	.001	.035	.000	.400	.400
VHS	r	.813^a^	.760^a^	.492^a^	-.445^b^	.584^a^	−.063	−.063
	p	.000	.000	.008	.018	.001	.750	.750
BNP	r	1	.880^a^	.366^b^	-.620^a^	.503^a^	−.068	−.068
	p		.000	.033	.000	.002	.702	.702
ANP	r	.880^a^	1	.322	-.510^a^	.413^b^	.132	.132
	p	.000		.064	.002	.015	.455	.455
Cholesterol	r	.366^b^	.322	1	−.276	.947^a^	.085	.085
	p	.033	.064		.115	.000	.634	.634
Triglyceride	r	−.068	.132	.085	.318	−.117	1	1.000^a^
	p	.702	.455	.634	.066	.510		.000
VLDL	r	−.068	.132	.085	.318	−.117	1.000^a^	1
	p	.702	.455	.634	.066	.510	.000	
HDL-C	r	-.620^a^	-.510^a^	−.276	1	-.557^a^	.318	.318
	p	.000	.002	.115		.001	.066	.066
LDL-C	r	.503^a^	.413^b^	.947^a^	-.557^a^	1	−.117	−.117
	p	.002	.015	.000	.001		.510	.510

^a^Correlation is significant at the 0.01 level, ^b^Correlation is significant at the 0.05 level

*MMVD* Myxomatous mitral valve disease, *LA/AO* Left atrium/aorta ratio, *LVIDd* Left ventricular end- diastolic diameter in diastole, *LVIDdN* Left ventricular end- diastolic diameter normalized for body weight, *VHS* Vertebral heart score. *HDL-C* High density lipoprotein, *LDL-C* Low-density lipoprotein, *VLDL* Very low-density lipoprotein, *BNP* B-type natriuretic peptide, *ANP* Atrial natriuretic peptide

## Discussion

BNP or NT-proBNP evaluation is the strongest indicator to confirm the diagnosis of congestive heart failure in humans [14]. These factors are also considered as prognostic parameters [15]. Several studies have reported that circulating BNP is increased in dogs with CHF due to MMVD. Our findings are consistent with those reported in previous studies. Also, in humans, BNP is an important diagnostic indicator for identifying patients with subclinical (asymptomatic) left ventricular dysfunction [14]. In this regard, different results have been reported in dogs with MMVD. Asano et al. described that plasma BNP was high only in dogs with moderate and severe CHF [16]. Häggström et al. reported that the BNP concentrations were increased (approximately twice) only in dogs with decompensated heart failure compared to normal and asymptomatic dogs [17]. However, MacDonald et al. reported that plasma BNP was high in dogs with moderate to severe MMVD in the absence of CHF. They also found that plasma BNP was higher in dogs with CHF due to MMVD [18]. In the present study, the mean serum BNP levels increase with the severity of MMVD. Our results showed that the mean serum BNP levels were significantly increased for all MMVD groups compared to control dogs.

In dogs, various studies have shown that circulating ANP levels increase with the severity of MMVD and the highest levels of ANP are seen in patients with stages C/D [4, 17, 19]. Our findings were consistent with those results. In our study, although the mean serum ANP levels in stage B2 (subclinical MMVD), C and D groups were significantly higher than the control group, the mean serum ANP concentrations did not differ significantly between the stage B1 (earliest subclinical MMVD) and control groups. Different reports have been presented in this regard. Some reports indicated that plasma ANP level was significantly higher for all MMVD groups than control dogs [4, 20], while a study in Cavalier King Charles Spaniels with MMVD found that plasma ANP levels were not increased significantly in asymptomatic dogs; but in dogs with decompensated heart failure, the mean ANP level was 3 to 7 times higher than control dogs [17]. These differences between the results of the present study with other reports may be due to differences in the BNP and ANP measurement methods, populations studied as well as the criteria used to classify the different stages of MMVD. Regarding the ANP measurement method, in our study, serum ANP was measured by a canine-specific ELISA kit, but in other studies, plasma ANP was determined using chemiluminescent enzyme immunoassay for human αANP [4], and radioimmunoassay [17].

Our results showed that increased serum BNP levels were observed in all cardiac patients except 3 patients in stage B1 group. However, in these 3 patients, BNP concentrations were within maximum normal values. Therefore, an increase in BNP level was observed in 87.5% (21/24) of patients. Although BNP concentrations were elevated in 100% of dogs with stages C, D and B2, high BNP was observed in 72.72% of dogs with stage B1. Regarding ANP, elevated ANP levels were found in only 27.27% (3/11) of patients in stage B1, while increased serum ANP levels were observed in 66.66% (4/6) of patients in the stage B2 group. However, in the C and D groups, 100% of patients had an increase in ANP. Therefore, increased ANP was also observed in 58.33% (14/24) of patients with MMVD. Because both the increase in BNP and the percentage of patients who showed an increase in serum BNP were significantly higher, serum BNP appears to have a greater diagnostic value than serum ANP in dogs with MMVD. In human, the indication for measuring BNP or NT-proBNP is detection of asymptomatic (occult or subclinical) heart disease. Our findings are consistent with the results of human research. Our results suggest that serum BNP may be a useful diagnostic marker for MMVD in dogs and can be used to determine the subclinical (asymptomatic) stages of B1 and B2 MMVD. Therefore, early diagnosis in asymptomatic patients can probably prevent the progression of the disease [14].

Echocardiographic variables and VHS are useful indices of disease severity in dogs with MMVD [2]. A previous study has found that only LA/AO ratio had a significant variable with the plasma ANP, NT-proBNP and NT-proANP [21]. However, in our study, serum natriuretic peptide (BNP and ANP) concentrations had a significant positive correlation with echocardiographic variables (LA/AO ratio, LVIDd, LVIDdN) and VHS. Mitral regurgitation is the earliest hemodynamic event. The progression of the disease and the increasing severity of valvular regurgitation generate a volume overload of the left heart, leading to left atrial and ventricular remodeling. In advanced stages of MMVD, associated volume overload promotes progressive valvular regurgitation, left atrial and left ventricular remodeling, atrial rupture, and CHF [22]. However, the difference between BNP and ANP secretion sources in normal and heart injury is still unclear, it seems that ventricles are the important source of natriuretic secretion, especially BNP, but ANP and BNP are mainly produced by the atria in healthy humans [23], and CHF patients [18]. In left ventricular dysfunction, when chronic hemodynamic pressure or volume overload occurs (such as ventricular hypertrophy), the ventricular myocytes undergo phenotypic modifications and re-express several fetal genes, including ANP and BNP [23, 24].

In humans, hyperlipidemia is a major risk factor for cardiovascular disease. In this regard, elevated LDL-C [25], and decreased HDL-C lead to atherogenic hyperlipidemia [26]. In the current study, mean HDL-C concentrations decreased and mean LDL-C concentrations increased in all dogs with acquired MMVD (B1, B2, C and D stages) compared to the control group. Additionally, the lowest mean of HDL-C and the highest mean of LDL-C were observed in the stage D group. Research has shown that elevated LDL-C in the blood leads to their accumulation as plugs in the vascular intimal. Fat particles, calcium and tissue macrophages play a major role in plug formation. However, plug formation reduces the elasticity and narrows the internal space of the arteries and eventually leads to blockage of the arteries or may even lead to rupture and eventually blood clots in the area [26]. HDL-C has anti-atherogenic effects. The anti-atherogenic effects of HDL-C are mediated through the reverse transport of cholesterol [27], and carriers of LDL- reducing potential oxidative proteins such as paraoxonase [28], and platelet activating factor acetyl hydrolase [29], as well as antioxidative and anti-inflammatory effects [30]. Therefore, it maintains endothelial integrity by using the above mechanisms [30]. HDL-C has also been shown to have antithrombotic and profibrinolytic effects [7]. These protective effects may be attenuated by lower blood HDL concentrations, which increases the risk of cardiovascular disease [30]. Although dogs appear to be resistant to atherosclerosis due to their heart structure (a well-developed coronary lateral circulation) [31], and lipoprotein composition [32], it has previously been described that arteriosclerotic changes and small myocardial infarcts are common in geriatric dogs with or without primary mitral regurgitation [33]. Also, Falk et al. reported that dogs with naturally MMVD had significantly more arterial changes and fibrosis in the myocardium than control dogs [34]. Therefore, Coronary arterial disease should also be considered in dogs with mitral regurgitation, which may contribute to reduced myocardial contractility [10]. However, the clinical and diagnostic value of blood lipoproteins in dogs with MMVD is still unclear. Our findings showed that dyslipidemia in dogs with MMVD (all stages) is mainly related to high LDL and low HDL.

We found a significant negative correlation between serum concentrations of BNP and HDL-C. This finding is similar to Takeuchi and Sata’s observation on human patients with cardiovascular disease [35]. Although, their result was not statistically significant. However, Lupattelli et al. reported a positive correlation between HDL-C and BNP in hyperlipemic humans [7]. Regarding LDL-C, our results showed that there is a significant positive correlation between BNP and LDL-C. In the present study, serum ANP, like BNP, had a negative correlation with HDL-C and a positive correlation with LDL-C. But it seems that high LDL-C is more involved in the pathogenesis of MMVD than low HDL-C. Since in the present study, LDL-C, but not HDL-C, had a significant positive correlation with echocardiographic variables, high LDL-C is more involved in the pathogenesis of MMVD than low HDL-C. However, further research is needed to determine the role of dyslipidemia on serum natriuretic peptides.

## Conclusions

Our study suggests that serum BNP is more valuable than serum ANP in dogs with MMVD. This study also indicates that patients with MMVD (stage B1 and B2 groups), who usually have no clinical symptoms, showed an increase in BNP along with a decrease in HDL-C and an increase in LDL-C, which are known to be risk factors for cardiovascular diseases in human. However, it seems that high LDL-C is more involved in the pathogenesis of MMVD than low HDL-C. Thus, periodic lipoprotein testing is recommended in high-risk patients, even if total cholesterol levels are normal. However, additional studies are needed to evaluate the role of dyslipidemia in the pathogenesis of MMVD.

## Data Availability

The data used and/or analysed during the current study are available from the corresponding author on reasonable request.

## Abbreviations

MMVD: Myxomatous mitral valve disease
ANP: Atrial natriuretic peptide
BNP: B-type natriuretic peptide
CHF: Congestive heart failure
ACVIM: American College of Veterinary Internal Medicine
LDL-C: Low-density lipoprotein cholesterol
HDL-C: High-density lipoprotein cholesterol
VLDL: Very low-density lipoprotein
VHS: Vertebral heart score
LVIDd: Left ventricular internal dimension-diastole
LVIDdN: LVIDd (cm)/weight (kg) ^0.294^
LA/Ao ratio: Left atrium/ aorta ratio
SE: Standard error
